# Retraction Note: Endoplasmic reticulum stress triggers Xanthoangelol-induced protective autophagy via activation of JNK/c-Jun Axis in hepatocellular carcinoma

**DOI:** 10.1186/s13046-020-01668-x

**Published:** 2020-08-19

**Authors:** Zichao Li, Luying Zhang, Mingquan Gao, Mei Han, Kaili Liu, Zhuang Zhang, Zhi Gong, Lifei Xing, Xianzhou Shi, Kui Lu, Hui Gao

**Affiliations:** 1 College of Life Sciences, Qingdao University, Qingdao, 266071 China; 2 Department of Pharmacology, School of Pharmacy, Qingdao University, Qingdao, 266021 China; 3 The Affiliated Cancer Hospital, School of Medicine, University of Electronic Science and Technology of China, Chengdu, 610041 Sichuan China; 4 China International Science and Technology Cooperation Base of Food Nutrition/Safety and Medicinal Chemistry, College of Biotechnology, Tianjin University of Science & Technology, Tianjin, 300457 China; 5 Northeast Yucai Bilingual School, Shenyang, 110164 China

**Retraction note to: J Exp Clin Cancer Res 38, 8 (2019)**

**https://doi.org/10.1186/s13046-018-1012-z**

The Editor-in-Chief has retracted this article [1] due to overlapping images with other articles, specifically:

- Figure 8d: the Beclin1 Vehicle and XAG (80 mg/kg) panels appear to overlap with the cleaved caspase-3 Vehicle and Exanthone (80 mg/kg/day) panels of Fig. 7c of [2].
- Figure 8d: the p-c-Jun Vehicle panel appears to overlap with the Beclin Vehicle panel of Fig. 7c of [2].
- Figure 3a: the Bel7402 XAG + 3-MA panel appears to be identical to the SMMC7721 Vehicle panel.
- Figure 3c: the Bel7402 XAG + Atg5 siRNA panel appears to be identical to the SMMC7721 Vehicle panel.
- Figure 3c: the SMMC7721 Atg5 siRNA panel appears to be similar to the SKOV3 Control and A2780 Control panels of Fig. 1e of [3], the Vehicle panel of Fig. 4b in [4] and the HT29 Control panel of Fig. 7b of [5]
- Figure 8d: the Vehicle CHOP, GRP78 and p-JNK panels appears to be similar to the Ki-67 EX(40 mg/kg) panel, the IL-18 Vehicle panel and the IL-18 EX(20 mg/kg) panel of [6].
- Figure 8d: the XAG(40 mg/kg) CHOP panel appears to be similar to the KI-67 EX(20 mg/kg) panel of [6].
- Figure 8d: the XAG(80 mg/kg) CHOP and GRP78 panels appear to be similar to the KI-67 Vehicle and the IL-18 EX(40 mg/kg) panels of [6].

The data reported in this article are therefore unreliable.

Hui Gao agrees to this retraction. Zichao Li, Luying Zhang, Mingquan Gao, Mei Han, Kaili Liu, Zhuang Zhang, Zhi Gong, Lifei Xing, Xianzhou Shi, Kui Lunot have responded to any correspondence from the publisher about this retraction.
